# Hepatic Tumor Stiffness Measured by Shear Wave Elastography Is Prognostic for HCC Progression Following Treatment With Anti-PD-1 Antibodies Plus Lenvatinib: A Retrospective Analysis of Two Independent Cohorts

**DOI:** 10.3389/fimmu.2022.868809

**Published:** 2022-06-09

**Authors:** Guosheng Yuan, Fuli Xie, Yangda Song, Qi Li, Rong Li, Xiaoyun Hu, Mengya Zang, Xiao Cheng, Guanting Lu, Jing Huang, Wenzhe Fan, Xiaoxiang Rong, Jian Sun, Jinzhang Chen

**Affiliations:** ^1^ Department of Infectious Diseases and Hepatology, Nanfang Hospital, Southern Medical University, Guangzhou, China; ^2^ Department of Oncology, Nanfang Hospital, Southern Medical University, Guangzhou, China; ^3^ Department of Hepatology, Zengcheng Branch, Nanfang Hospital, Southern Medical University, Guangzhou, China; ^4^ Department of Interventional Oncology, The First Affiliated Hospital of Sun Yat-Sen University, Guangzhou, China

**Keywords:** hepatocellular carcinoma, pd-1, lenvatinib, shear wave elastography, stiffness

## Abstract

**Background:**

The clinical significance of liver stiffness (LS) measured by shear wave elastography (SWE) in programmed cell death protein-1 (PD-1) inhibitors treated advanced hepatocellular carcinoma (HCC) patients remains unknown. This study aimed to explore the prognostic value of baseline LS by SWE prior to PD-1 inhibitor treatment in combination with lenvatinib.

**Methods:**

We retrospectively evaluated patients (n=133) with HCC who received anti-PD-1 antibodies plus lenvatinib at two high-volume medical centres, between January 2020 and June 2021. Univariate and multivariate logistic regression analysis were used to develop a novel nomogram. RNA sequencing and immunohistochemical staining were used to assess the heterogeneity of biological and immune characteristics associated with tumor stiffness.

**Results:**

The objective response rate (ORR) and disease control rate (DCR) of the whole population were 23.4% and 72.2%, respectively. A LS value of the baseline tumorous foci of 19.53 kPa had the maximum sum of sensitivity and specificity, making it the optimal cut-off value for predicting PD-1 inhibitor efficacy. The nomogram comprised baseline tumor LS and albumin-bilirubin grade (ALBI), which provided favorable calibration and discrimination in the training dataset with an AUC of 0.840 (95%CI: 0.750-0.931) and a C-index of 0.828. Further, it showed acceptable discrimination in the validation cohort, with an AUC of 0.827 (95%CI: 0.673-0.980) and C-index of 0.803. The differentially expressed genes enriched in high stiffness tumors were predominantly associated with metabolic pathways, while those enriched in low stiffness tumors were related to DNA damage repair. Furthermore, patients with high stiffness tumors had a relatively lower infiltration of immune cells and histone deacetylase pathway inhibitors were identified as candidate drugs to promote the efficacy of immunotherapy.

**Conclusions:**

Baseline LS value of tumorous foci by SWE—that is, before administration of a PD-1 inhibitor in combination with lenvatinib—is a convenient predictor of PD-1 inhibitor efficacy in patients with advanced HCC, which has potential to be used for pretreatment stratification to optimize treatment of advanced HCC.

## Highlights

- Question: Is hepatic tumor stiffness measured by shear wave elastography (SWE) useful in predicting HCC progression following treatment with Anti-PD-1 antibodies plus Lenvatinib?
- Pertinent Findings: Baseline tumor LS by SWE, before anti-PD-1 in combination with lenvatinib, is a convenient predictor of tumor progression in patients with advanced HCC through a retrospective analysis of two independent cohorts.
- Implications for Patient Care: Our data shed light on the application of tumor LS in predicting HCC progression, which might guide the development of rational strategies for use of anti-PD-1 in combination antiangiogenic agents, ultimately benefiting a broader range of patients.

## Introduction

Despite the introduction of new targeted therapies for advanced hepatocellular carcinoma (HCC) over the last few decades, the prognosis of patients with advanced HCC remains poor as these treatment strategies are not wholly effective (1–3). In recent years, inhibitors of the programmed cell death-1 (PD-1)/programmed cell death ligand-1 (PD-L1) pathway have received much attention as HCC immunotherapies (4–6). However, previous studies showed that fewer than 20% of advanced HCC patients achieved an objective tumor response in anti-PD-1 monotherapy, indicating that combination regimens—e.g., anti-PD-1 with an antiangiogenic therapy—might be better options for systemic treatment (7–9). Indeed, the Imbrave150 study has demonstrated a new combination regimen (atezolizumab plus bevacizumab, also known as “T+A” strategy) to be superior to sorafenib (a kinase inhibitor), showing a 42% reduction in risk of death, resulting in the “T+A” strategy to be the recommended first-line treatment for patients with advanced HCC globally (10). In addition, the RESCUE trial showed that combined camrelizumab with apatinib was also promising, with an objective response rate (ORR) of 34.3% and a disease control rate (DCR) of 77.1% in advanced HCC patients when used as a first-line treatment (11). Nevertheless, in the above studies, only a fraction of patients benefited from anti-PD-1/PD-L1 antibodies in combination with an antiangiogenic therapy. Therefore, it’s urgently needed to identify factors that can predict a curative effect to define which patients with advanced HCC are most likely to benefit from therapy with anti-PD-1 antibodies in combination with antiangiogenic therapy.

More than 80% of HCC cases arise in the cirrhotic liver (12) and the degree of liver fibrosis has been reported to be a negative prognostic factor for sorafenib therapy (13). With recent advances in ultrasound technology, various elastography techniques have been found to be effective in staging liver fibrosis, among which shear wave elastography (SWE)—which uses the supersonic shear imaging technique—is capable of predicting overall survival in patients after radiofrequency ablation for HCC (14). In addition, previous studies reported that liver stiffness (LS) values measured by SWE achieved better sensitivity and specificity than those measured by transient elastography (TE), the aspartate aminotransferase (AST) to Platelet Ratio Index, or the Fibrosis 4 score in patients with chronic hepatitis B (15–17). However, the clinical implications of baseline LS values by SWE in patients with advanced HCC who are treated with anti-PD-1/PD-L1 or in those treated with anti-PD-1/PD-L1 in combination with an antiangiogenic regimen have not been explored.

Herein, we aimed to investigate the clinical significance of tumor-LS by SWE in anti-PD-1 antibodies in combination with lenvatinib treated HCC patients. We demonstrated that the tumor LS value, as measured by SWE before combination treatment with anti-PD-1 antibodies plus lenvatinib, was a convenient predictor of tumor progression in patients with advanced HCC. We further explored the heterogeneity of biological and immune characteristics associated with tumor stiffness in 9 HCC tissue samples across two groups having different tumor LS values (a high LS group and a low LS group), using RNA sequencing and immunohistochemical (IHC) staining. Our data shed light on the application of tumor LS for predicting HCC progression, which might guide the development of rational regimens using anti-PD-1 antibodies in combination with an antiangiogenic therapy, to ultimately benefit a greater range of patients.

## Materials and Methods

### Patients

This retrospective study included patients with HCC who underwent SWE before anti-PD-1 antibodies in combination with lenvatinib therapy, starting from June 1, 2020 to May 31, 2021 at Nanfang Hospital, Southern Medical University, and from January 1, 2020 to June 30, 2021 at the First Affiliated Hospital of Sun Yat-Sen University. All patients were aged ≥18 years with HCC diagnosed either by two imaging modalities or by biopsy according to the diagnostic criteria (18). This study was designed and performed according to the Declaration of Helsinki and was approved by the Medical Ethics Committee of Nanfang hospital and the First Affiliated Hospital of Sun Yat-Sen University. Written informed consent was obtained from each patient to retrospectively review and report on their medical records.

Patients with the following characteristics were excluded: (1) those who accepted locoregional therapy before treatment or during follow-up; (2) those with Child-Pugh C liver function; (3) those who were coinfected with hepatitis A, C, or D virus or HIV; (4) women who were pregnant or breastfeeding; (5) those whose Eastern Cooperative Oncology Group performance status was over 3; and (6) those who were lost to follow-up other than death within 3 months after treatment.

**Figure 1:**
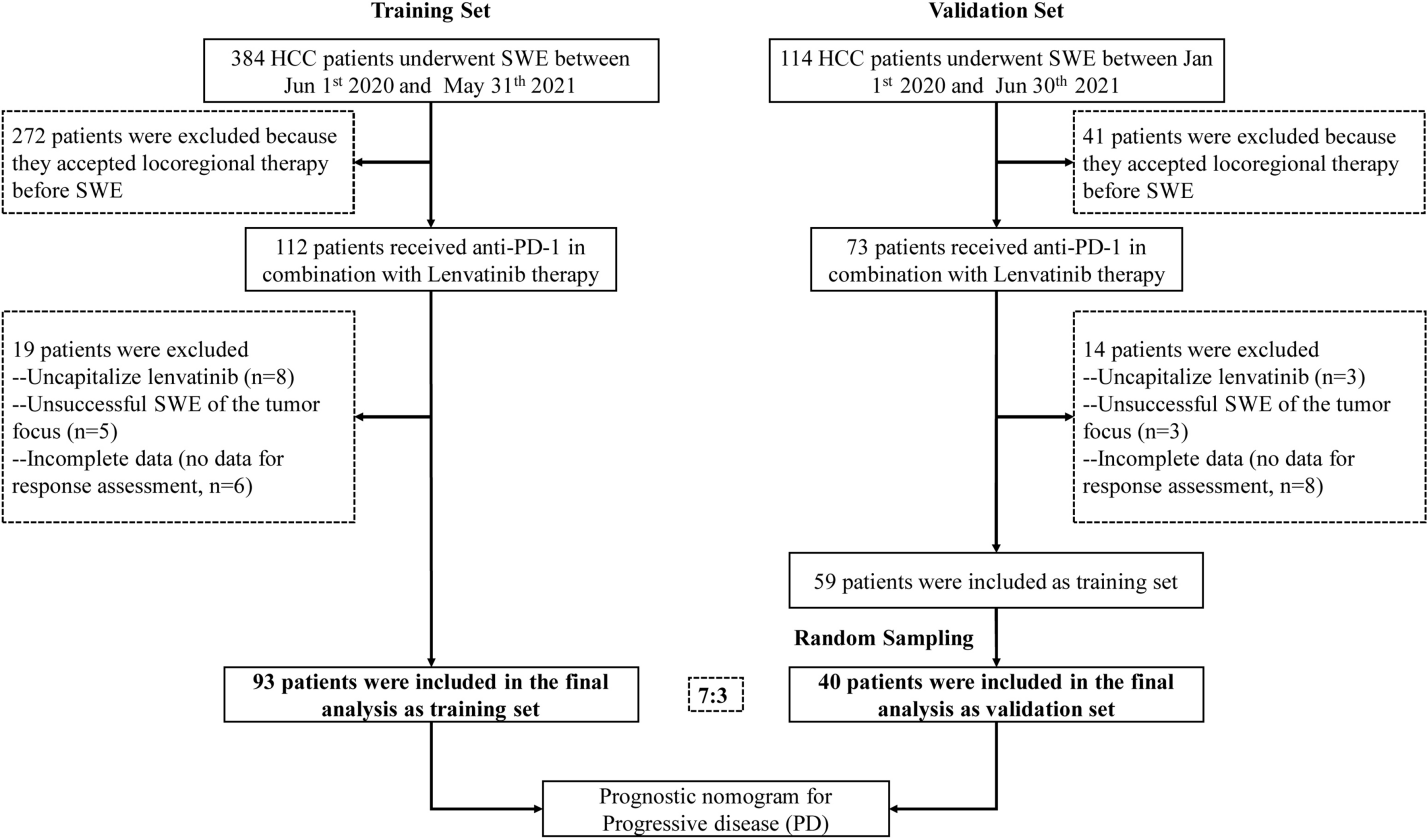
Patient recruitment flowchart.

Ultimately, 93 patients with complete data were included in the training set. Furthermore, the validation cohort of 40 patients (7:3), who satisfied the inclusion and exclusion criteria, was selected by a random extraction of HCC patients treated at the First Affiliated Hospital of Sun Yat-Sen University. A flowchart showing patient selection is depicted in **Figure 1**.

### LS Measurements Using SWE

LS values were measured in both tumorous foci and adjacent normal liver regions before the first dose of a PD-1 inhibitor using the ARFI imaging technology implemented on a Siemens Acuson S2000 ultrasound system (Siemens AG, Erlangen, Germany) at Nanfang hospital and on Aixplorer (SuperSonic Imagine, France) at the First Affiliated Hospital of Sun Yat-Sen University. Tumorous foci in the participants were previously identified as HCC by two imaging modalities or by biopsy, while the adjacent normal liver tissues were selected by the operator’s preference. The physician who performed SWE had 17 years of experience. The SWE protocol was performed according to the European Federation of Societies for Ultrasound in Medicine and Biology and World Congress for Ultrasound in Medicine and Biology guidelines (19, 20). Results expressed in m/s from the virtual tissue quantification were converted to the Young’s modulus and expressed in kiloPascals (kPa). LS measurements were considered reliable if they achieved an interquartile range interval/median ratio (IQR/M) of ≤0.3.

### Treatment and Assessment

The dosage of anti-PD-1 therapy has been described in our previous studies (7, 21, 22); briefly, one of the following regimens was selected for each patient: toripalimab, 3 mg/kg body weight or 240 mg, once every 2 weeks by IV; camrelizumab, 200 mg, q2/3w, IV; or sintilimab, 200 mg, q3w, IV. The dosage of the antiangiogenic therapy lenvatinib was 8 mg once per day orally.

Demographic, clinical, and laboratory data were collected from all patients prior to initiating PD-1 inhibitor therapy. Data included the patient’s age, gender, α-fetoprotein, alanine aminotransferase, aspartate transaminase, prothrombin time, albumin level, platelet count, total bilirubin, Barcelona Clinic Liver Cancer (BCLC) stage, Eastern Cooperative Oncology Group (ECOG) performance, Child-Pugh score, tumor size, number, vascular invasion, and extrahepatic metastasis. The albumin-bilirubin (ALBI) score was calculated for each patient by the following formula: ALBI score = (log_10_ bilirubin × 0.66) + (albumin × −0.085), where bilirubin is in µmol/L and albumin in g/L.

Patients underwent computed tomography or magnetic resonance imaging at baseline, at 6-12 weeks after treatment initiation, and about every 3 months thereafter. Treatment-related Aes were recorded at every visit according to the US National Cancer Institute Common Terminology Criteria for Adverse Events (CTCAE v5.0). The modified response evaluation criteria in solid tumors (mRECIST) were used for tumor response evaluation (23) as follows: (1) complete response (CR), where the target lesions disappeared according to enhanced imaging in the arterial phase; (2) partial response (PR), where the diameter of the target lesions was reduced by ≥30% according to enhanced imaging in the arterial phase; (3) stable disease (SD), where the diameter of the target lesions was not reduced to threshold of PR, but it also did not increase past that in progressive disease (PD); (4) PD, which was marked by a total increase of ≥20% in the diameter of the target lesions according to enhanced imaging in the arterial phase compared with the baseline value, or if new lesions appeared.

### Development and Validation of Combination Nomogram

Clinical characteristics were selected through univariate and multivariate logistic regression analysis and a nomogram was built based on the independent risk factors in the multivariate analysis. Details were also described in our previously report (22).

### RNA Sequencing of Samples From Patients With HCC who Were Diagnosed in Nanfang Hospital

A total of 9 patients with HCC were recruited for RNA sequencing, of whom 5 had a high stiffness tumor (LS >19.53 kPa) and 4 had a low stiffness tumor (LS ≤19.53 kPa). All patients received a fine needle aspiration biopsy. The biopsy specimens were divided for both paraffin embedding and storing in RNA*later* ^®^ (Qiagen). MGISEQ-2000 was used to generate transcriptome data.

### Immunohistochemistry

IHC was performed in accordance with previous reports (24, 25). Briefly, the 5-μm HCC sections embedded in paraffin were deparaffinized and treated with hydrogen peroxide to quench endogenous peroxidase activity. The following primary antibodies were used: Rabbit monoclonal [SP7] anti-CD3 antibody (100 μl; Abcam), and Rabbit monoclonal [CAL66] anti-CD8 antibody (100 μl; Abcam). All histopathological analyses were performed by an experienced histopathologist (with 21 years of experience), who was blinded to all clinical data. The CD3 and CD8 cell infiltrations were accessed by calculating the proportion of positive stained cells as follow: no staining, 1+; weak staining, 2+; moderate staining, 3+; strong staining, 4+; and intense staining, 5+. Accordingly, the score of 1+ and 2+ was defined as low expression while the other scores were defined as high expression.

### Public Transcriptome Data Collection and Preprocessing

We accessed public transcriptome data of HCC patients from the Gene Expression Omnibus and TCGA databases. For samples from the TCGA database, we downloaded level three “HTSeq-Counts” data from the UCSC Xena website (https://xenabrowser.net/). The “voom” algorithm was used to transform RNA sequencing data, as previously described (26). For the GSE109211 dataset, we downloaded the “Series Matrix File(s),” containing normalized transcriptome data.

### Development of a tumor Stiffness–Related Gene Expression Signature

To establish a tumor stiffness–related gene expression signature, we firstly performed differential expression gene (DEG) analysis between high and low stiffness HCC samples using the “DESeq2” package. The significance criterion for DEGs was set as an absolute log2FC value >1.0 and an adjusted *P* value <0.05. The top 100 genes with the largest absolute log2FC values in the log2FC value >1 group and the top 50 genes with the largest absolute log2FC values in the log2FC value <−1 group were separately selected to develop a tumor stiffness–related gene expression signature. The tumor stiffness of each sample was predicted using the nearest template prediction (NTP, Gene Pattern) algorithm based on our developed tumor stiffness–related gene expression signature.

### Biological Processes and Tumor Microenvironment Characteristics Analysis

A biological process analysis was performed using gene set variation analysis (GSVA) based on the gene set files of “c2.cp. kegg. v6.2. symbols.” The immune infiltration estimation was conducted using the “Microenvironment Cell Populations-counter (MCP-counter)” method by applying the “IOBR” R packages.

### Connectivity Map Analysis

Cmap analysis was performed to identify effective candidate compounds following the instructions provided by the Cmap website (https://clue.io/).

### Statistical Analysis

Statistical analyses were performed using SPSS 22.0 software (SPSS Inc., Chicago, IL) and R software (version 3.6.2, http://www.Rproject.org). Data were expressed as counts and percentages for categorical variables, such as those in baseline characteristics, radiological tumor response, and adverse events (AEs). Qualitative differences between subgroups were analyzed using χ² tests or Fisher’s exact test for categorical parameters. For analyzing the performance ability of quantitative LS to predict treatment outcome, the area under the receiver operator characteristic curve (AUROC) was calculated. AUROCs were compared by the Delong test by using the “pROC” package and the DCA curves were plotted by using the “RMDA” package. Sensitivity, specificity, positive likelihood ratio, and negative likelihood ratio of several cut-off values of LS levels were calculated to explore the best cut-off value in predicting treatment efficacy. Univariate and multivariate logistic regression analyses were performed to assess factors related to treatment outcomes. All statistical analyses were based on 2-tailed hypothesis tests with a significance level of *P*<0.05.

## Results

### Patient Characteristics

For the analysis, a total of 93 and 40 patients (7:3) were included in the training and validation sets, respectively. **Table 1** shows the clinical characteristics of these patients at baseline. The proportion of patients with BCLC stage C disease was 77.4% (72/93) and 72.5% (29/40), and the proportion of patients with ALBI grade 1 were 55.9% (52/93) and 60.0% (24/40) in the training and validation sets, respectively. The LS values, as determined by SWE, of the tumorous foci and adjacent normal liver tissue in the training set were 17.70 ± 7.78 kPa and 13.96 ± 8.26 kPa, respectively, and the corresponding values in the validation set were 16.87 ± 6.68 kPa and 15.16 ± 8.44 kPa. Among the 133 patients, 96 (72.2%) achieved tumor control (CR + PR + SD), while 37 patients (27.8%) exhibited progressive disease (PD) (**Table 2**). The ORR was 23.4% and the DCR was 72.2% (**Table 2**).

**Table 1:** Baseline patient characteristics in the training and validation sets.

Characteristics	All patients (n = 133)	Training set (n = 93)	Validation set (n = 40)	*P* value^#^
Age (years)	53 ± 12	54 ± 12	51 ± 11	0.243
Gender				0.232
Men, n (%)	116 (87.2)	79 (84.9)	37 (92.5)	
Women, n (%)	17 (12.8)	14 (15.1)	3 (7.5)	
AFP (ng/ml)				0.807
<20	57 (42.9)	39 (41.9)	18 (45.0)	
20-400	24 (18.0)	16 (17.2)	8 (20.0)	
>400	52 (39.1)	38 (40.9)	14 (35.0)	
ALT (U/L)	29.0 (5.00, 1048.00)	26.00 (5.00, 1048.00)	35.00 (11.00, 125.00)	0.802
AST (U/L)	40.50 (9.00, 723.00)	38.00 (9.00, 723.00)	52.00(18.00, 324.00)	0.249
PT (s)	11.83 ± 1.62	11.88 ± 1.80	11.69 ± 1.09	0.538
ALB (g/L)	36.32 ± 5.59	36.00 ± 5.66	37.06 ± 5.36	0.287
PLT (10^9^/L)	160.9 ± 84.8	159.92 ± 88.90	163.15 ± 75.58	0.970
TBIL (µmol/L)	17.80 ± 10.60	16.91 ± 9.59	19.59 ± 12.63	0.183
BCLC				0.543
B, n (%)	32 (24.1)	21 (22.6)	11 (27.5)	
C, n (%)	101 (75.9)	72 (77.4)	29 (72.5)	
ECOG performance^*^				0.973
0, n (%)	93 (69.9)	65 (69.9)	28 (70.0)	
1, n (%)	36 (27.1)	25 (26.9)	11 (27.5)	
2, n (%)	4 (3.0)	3 (3.2)	1 (2.5)	
3, n (%)	0	0	0	
Child-Pugh grade				0.877
A	114 (85.7)	80 (86.0)	34 (85.0)	
B	19 (14.3)	13 (14.0)	6 (15.0)	
ALBI	-2.29 ± 0.53	-2.29 ± 0.53	−2.29 ± 0.51	0.984
ALBI grade				0.662
1, n (%)	76 (57.1)	52 (55.9)	24 (60.0)	
2, n (%)	57 (42.9)	41 (44.1)	16 (40.0)	
Tumor number				0.773
<3 nodules, n (%)	84 (63.2)	58 (62.4)	26 (65.0)	
≥3 nodules, n (%)	49 (36.8)	35 (37.6)	14 (35.0)	
Tumor size (cm)	7.41 ± 4.30	7.43 ± 4.38	7.35 ± 4.16	0.922
Embolus				0.215
Absent, n (%)	74 (55.6)	55 (59.1)	19 (47.5)	
Present, n (%)	59 (44.4)	38 (40.9)	21 (52.5)	
Extrahepatic metastasis				0.845
Absent, n (%)	105 (78.9)	73 (78.5)	32 (80.0)	
Present, n (%)	28 (21.1)	20 (21.5)	8 (20.0)	
LS value of tumorous foci by SWE (kPa)	17.49 ± 7.47	17.70 ± 7.78	16.87 ± 6.68	0.557
LS value of adjacent normal liver tissue by SWE (kPa)	14.32 ± 8.30	13.96 ± 8.26	15.16 ± 8.44	0.409

AFP, α-fetoprotein; ALB, albumin; ALBI, albumin-bilirubin; ALT, alanine aminotransferase; AST, aspartate aminotransferase; BCLC, Barcelona Clinic Liver Cancer; ECOG, Eastern Cooperative Oncology Group; LS, liver stiffness; PD-1, programmed cell death protein 1; PLT, platelet count; SWE, shear-wave elastography; PT, prothrombin time; TBIL, total bilirubin.

ALBI score = (log_10_ bilirubin × 0.66) + (albumin × −0.085). ^*^Fisher’s exact test, others used χ² tests. ^#^Comparison between the validation and training sets.

**Table 2:** Tumor responses.

Tumor response, n (%)	All patients (n = 133)	Training set (n = 93)	Validation set (n = 40)
Complete response (CR)	1 (0.8)	1 (1.1)	0
Partial response (PR)	30 (22.6)	19 (20.4)	11 (27.5)
Stable disease (SD)	65 (48.9)	46 (49.5)	19 (47.5)
Progressive disease (PD)	37 (27.8)	27 (29.0)	10 (25.0)
ORR (CR + PR)^*^	31 (23.4)	20 (21.5)	11 (27.5)
DCR (CR + PR + SD)^#^	96 (72.2)	66 (71.0)	30 (75.0)

DCR, disease control rate; ORR, objective response rate.

^*^Pearson χ² = 0.562, P = 0.453 (ORR comparison between the validation and training sets).

^#^Pearson χ² = 0.226, P = 0.634 (DCR comparison between the validation and training sets).

### Safety Analysis

All recorded treatment-related AEs are shown in **Table 3**. Forty-nine patients (49/133, 36.8%) experienced at least 1 AE during treatment with a PD-1 inhibitor in combination with lenvatinib. The 5 most frequent types of AEs were hepatitis (42/133, 31.6%), abdominal pain (36/133, 27.1%), thrombocytopenia (36/133, 27.1%), diarrhea (32/133, 24.1%), and hypothyroidism (31/133, 23.3%). A dose delay due to AEs was required in 10 patients. Steroids or immunosuppressive drugs were used to treat AEs in 14 patients, and no patients stop treatment due to AEs.

**Table 3:** Treatment-related adverse events in the training and validation sets.

Adverse Event	All patients (n = 133)		Training set (n = 93)		Validation set (n = 40)	
	All Grades, n (%)	Grades 3/4, n (%)	All Grades, n (%)	Grades 3/4, n (%)	All Grades, n (%)	Grades 3/4, n (%)
Hepatitis*	42 (31.6)	4 (3.0)	33 (35.5)	2 (2.2)	9 (22.5)	2 (5.0)
Abdominal pain	36 (27.1)	3 (2.3)	29 (31.2)	3 (3.2)	7 (17.5)	0
Thrombocytopenia	36 (27.1)	1 (0.8)	25 (26.9)	0	11 (27.5)	1 (2.5)
Diarrhea	32 (24.1)	4 (3.0)	23 (24.7)	3 (3.2)	9 (22.5)	1 (2.5)
Hypothyroidism	31 (23.3)	4 (3.0)	25 (26.9)	2 (2.2)	6 (15.0)	2 (5.0)
Hypertension	30 (22.6)	3 (2.3)	22 (23.7)	0	8 (20.0)	3 (7.5)
Headache	29 (21.8)	2 (1.5)	19 (20.4)	2 (2.2)	10 (25.0)	0
Fatigue	25 (18.8)	0	18 (19.4)	0	7 (17.5)	0
Proteinuria	24 (18.0)	3 (2.3)	18 (19.4)	1 (1.1)	6 (15.0)	2 (5.0)
Rash	22 (16.5)	4 (3.0)	19 (20.4)	3 (3.2)	3 (7.5)	1 (2.5)
Leukopenia	14 (10.5)	0	11 (11.8)	0	3 (7.5)	0
Vomiting	11 (8.3)	1 (0.8)	9 (9.7)	1 (1.1)	2 (5.0)	0
Hoarseness	6 (4.5)	0	4 (4.3)	0	2 (5.0)	0
Dental ulcer	3 (2.3)	0	3 (3.2)	0	0	0

There was no grade 5 adverse event (death) in any patient.

* Hepatitis was detected by Alanine aminotransferase (ALT) or aspartate aminotransferase (AST) increase.

### Correlation Between Baseline Variables and PD

To further evaluate baseline variables that might predict PD, a logistic regression analysis was conducted in the training set. A univariate regression analysis identified the following 2 factors as associated with PD: ALBI grade 1 (odds ratio [OR] 0.124, 95% CI 0.044-0.354, *P*<0.001) and LS value of tumorous foci by SWE (kPa) (OR 1.139, 95% CI 1.063-1.222, *P*<0.001). We then entered these significant factors into a multivariate analysis and found that along with an ALBI grade of 1 (OR 0.107, 95% CI 0.032-0.359, *P*<0.001), the baseline LS value of the tumor (OR 1.148, 95% CI 1.064-1.239, *P*<0.001) was an independent predictive factor for PD (**Table 4**).

**Table 4:** Univariate and multivariate logistic regression analyses of baseline variables predicting PD in training set (n = 93).

Factors	Univariate			Multivariate		
	OR	95% CI	*P* Value	OR	95% CI	*P* Value
Age	0.981	0.945-1.018	0.308			
Gender: Male/Female	1.439	0.434-4.773	0.552			
AFP (ng/mL):			0.514			
<20^*^						
20-400	2.000	0.569-7.028	0.280			
>400	1.538	0.560-4.230	0.404			
ALT (U/L)	0.998	0.992-1.005	0.629			
AST (U/L)	0.999	0.993-1.005	0.773			
PT (s)	1.053	0.814-1.362	0.694			
ALB (g/L)	1.009	0.932-1.093	0.829			
PLT (10^9^/L)	1.001	0.997-1.006	0.592			
TBIL (µmol/L)	1.002	0.957-1.050	0.923			
BCLC stage: B/C	0.769	0.271-2.184	0.622			
ECOG performance:			0.909			
0^*^						
1	1.229	0.452-3.342	0.687			
2	1.306	0.111-15.299	0.832			
Child-Pugh: B/A	1.101	0.308-3.936	0.882			
ALBI grade:1/2	0.124	0.044-0.354	**<0.001**	0.107	0.032-0.359	**<0.001**
Tumor number: ≥3/<3	1.857	0.746-4.623	0.183			
Tumor size (cm)	0.938	0.841-1.046	0.252			
Embolus: Present/Absent	1.568	0.615-3.998	0.347			
Extrahepatic metastasis: Present/Absent	1.061	0.360-3.132	0.914			
LS value of tumor by SWE (kPa)	1.139	1.063-1.222	**<0.001**	1.148	1.064-1.239	**<0.001**
LS value of adjacent normal liver tissue by SWE (kPa)	0.999	0.946-1.055	0.962			

AFP, α-fetoprotein; ALB, albumin; ALBI, albumin-bilirubin; ALT, alanine aminotransferase; AST, aspartate aminotransferase; ECOG, Eastern Cooperative Oncology Group; LS, liver stiffness; PD, Progressive disease; PLT, platelet count; PT, prothrombin time; TBIL, total bilirubin; SWE, shear-wave elastography.

*Used as the reference category. Bold values are values with statistical differences (P value<0.05).

### Performance Ability of The Baseline LS Value in Predicting PD

**Figure 2:**
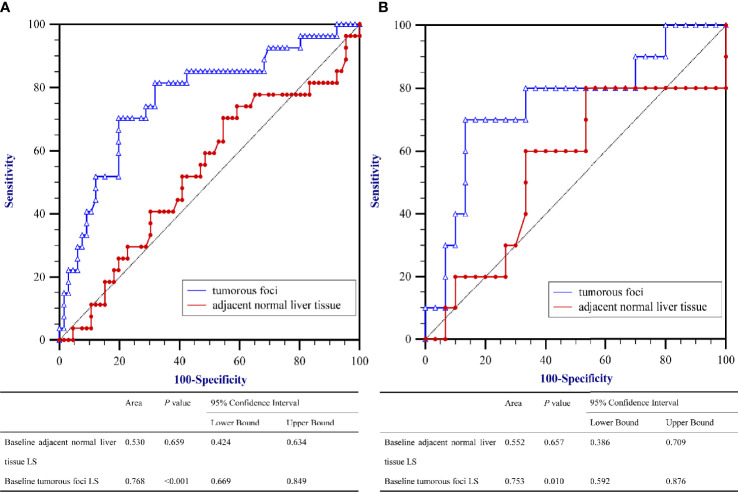
Area under the receiver operating characteristics curves (AUROC) for predicting Progressive Diseases (PD) after therapy with anti–programmed cell death protein 1 (PD-1) plus lenvatinib using baseline liver stiffness (LS) values of tumorous foci. **(A)** The training set (n = 93) and **(B)** the validation set (n = 40).

To evaluate the correlation between PD and the baseline LS value, as determined by SWE prior to initiation of PD-1 inhibitor–based therapy, AUROCs were calculated (**Figure 2**). AUROCs of the LS values of the tumorous foci were higher than those of the adjacent normal liver tissue in both the training and validation sets (0.768 and 0.753, respectively). **Table 5** shows the sensitivity and specificity for predicting PD from the LS value of the tumorous foci in the training set. The sum of sensitivity and specificity was highest when the cut-off value was 19.53 kPa. Therefore, 19.53 kPa was adopted as the optimal cut-off value for LS of the tumorous foci in the following analyses.

**Table 5:** Performance of baseline LS value in predicting PD in training set (n = 93).

Cut-off values	Sensitivity (%)	95% CI	Specificity (%)	95% CI	+LR	-LR
>=3.97	100	87.2 - 100.0	0	0.0 - 5.4	1	
>3.97	100	87.2 - 100.0	1.52	0.04 - 8.2	1.02	0
>5.07	100	87.2 - 100.0	3.03	0.4 - 10.5	1.03	0
>5.15	100	87.2 - 100.0	4.55	0.9 - 12.7	1.05	0
>5.39	100	87.2 - 100.0	6.06	1.7 - 14.8	1.06	0
>7.21	100	87.2 - 100.0	7.58	2.5 - 16.8	1.08	0
>7.66	96.3	81.0 - 99.9	7.58	2.5 - 16.8	1.04	0.49
>8.17	96.3	81.0 - 99.9	9.09	3.4 - 18.7	1.06	0.41
>8.27	96.3	81.0 - 99.9	10.61	4.4 - 20.6	1.08	0.35
>9.08	96.3	81.0 - 99.9	12.12	5.4 - 22.5	1.1	0.31
>9.19	96.3	81.0 - 99.9	13.64	6.4 - 24.3	1.12	0.27
>9.51	96.3	81.0 - 99.9	15.15	7.5 - 26.1	1.13	0.24
>9.61	96.3	81.0 - 99.9	16.67	8.6 - 27.9	1.16	0.22
>9.72	96.3	81.0 - 99.9	18.18	9.8 - 29.6	1.18	0.2
>10.16	96.3	81.0 - 99.9	19.7	10.9 - 31.3	1.2	0.19
>10.83	92.59	75.7 - 99.1	19.7	10.9 - 31.3	1.15	0.38
>10.94	92.59	75.7 - 99.1	24.24	14.5 - 36.4	1.22	0.31
>11.11	92.59	75.7 - 99.1	25.76	15.8 - 38.0	1.25	0.29
>11.17	92.59	75.7 - 99.1	27.27	17.0 - 39.6	1.27	0.27
>11.29	92.59	75.7 - 99.1	28.79	18.3 - 41.3	1.3	0.26
>11.52	92.59	75.7 - 99.1	30.3	19.6 - 42.9	1.33	0.24
>11.88	88.89	70.8 - 97.6	31.82	20.9 - 44.4	1.3	0.35
>12.2	85.19	66.3 - 95.8	31.82	20.9 - 44.4	1.25	0.47
>12.54	85.19	66.3 - 95.8	33.33	22.2 - 46.0	1.28	0.44
>12.6	85.19	66.3 - 95.8	34.85	23.5 - 47.6	1.31	0.43
>12.76	85.19	66.3 - 95.8	36.36	24.9 - 49.1	1.34	0.41
>13.02	85.19	66.3 - 95.8	37.88	26.2 - 50.7	1.37	0.39
>13.1	85.19	66.3 - 95.8	39.39	27.6 - 52.2	1.41	0.38
>13.23	85.19	66.3 - 95.8	40.91	29.0 - 53.7	1.44	0.36
>13.29	85.19	66.3 - 95.8	42.42	30.3 - 55.2	1.48	0.35
>13.36	85.19	66.3 - 95.8	43.94	31.7 - 56.7	1.52	0.34
>13.74	85.19	66.3 - 95.8	45.45	33.1 - 58.2	1.56	0.33
>14.39	85.19	66.3 - 95.8	46.97	34.6 - 59.7	1.61	0.32
>14.92	85.19	66.3 - 95.8	48.48	36.0 - 61.1	1.65	0.31
>15.05	85.19	66.3 - 95.8	50	37.4 - 62.6	1.7	0.3
>15.23	85.19	66.3 - 95.8	51.52	38.9 - 64.0	1.76	0.29
>15.32	85.19	66.3 - 95.8	53.03	40.3 - 65.4	1.81	0.28
>15.34	85.19	66.3 - 95.8	54.55	41.8 - 66.9	1.87	0.27
>15.35	85.19	66.3 - 95.8	56.06	43.3 - 68.3	1.94	0.26
>15.46	85.19	66.3 - 95.8	57.58	44.8 - 69.7	2.01	0.26
>15.5	81.48	61.9 - 93.7	57.58	44.8 - 69.7	1.92	0.32
>15.87	81.48	61.9 - 93.7	59.09	46.3 - 71.0	1.99	0.31
>16.22	81.48	61.9 - 93.7	60.61	47.8 - 72.4	2.07	0.31
>16.29	81.48	61.9 - 93.7	62.12	49.3 - 73.8	2.15	0.3
>16.64	81.48	61.9 - 93.7	63.64	50.9 - 75.1	2.24	0.29
>16.85	81.48	61.9 - 93.7	66.67	54.0 - 77.8	2.44	0.28
>17.13	81.48	61.9 - 93.7	68.18	55.6 - 79.1	2.56	0.27
>17.14	74.07	53.7 - 88.9	68.18	55.6 - 79.1	2.33	0.38
>18.09	74.07	53.7 - 88.9	69.7	57.1 - 80.4	2.44	0.37
>18.57	74.07	53.7 - 88.9	71.21	58.7 - 81.7	2.57	0.36
>18.6	70.37	49.8 - 86.2	71.21	58.7 - 81.7	2.44	0.42
>19.02	70.37	49.8 - 86.2	72.73	60.4 - 83.0	2.58	0.41
>19.2	70.37	49.8 - 86.2	75.76	63.6 - 85.5	2.9	0.39
>19.35	70.37	49.8 - 86.2	77.27	65.3 - 86.7	3.1	0.38
>19.51	70.37	49.8 - 86.2	78.79	67.0 - 87.9	3.32	0.38
**>19.53** *	**70.37**	**49.8 - 86.2**	**80.3**	**68.7 - 89.1**	**3.57**	**0.37**
>19.81	66.67	46.0 - 83.5	80.3	68.7 - 89.1	3.38	0.42
>19.88	62.96	42.4 - 80.6	80.3	68.7 - 89.1	3.2	0.46
>19.94	59.26	38.8 - 77.6	80.3	68.7 - 89.1	3.01	0.51
>20.44	51.85	31.9 - 71.3	80.3	68.7 - 89.1	2.63	0.6
>20.75	51.85	31.9 - 71.3	84.85	73.9 - 92.5	3.42	0.57
>21.07	51.85	31.9 - 71.3	87.88	77.5 - 94.6	4.28	0.55
>22.03	48.15	28.7 - 68.1	87.88	77.5 - 94.6	3.97	0.59
>22.87	44.44	25.5 - 64.7	87.88	77.5 - 94.6	3.67	0.63
>23.02	40.74	22.4 - 61.2	89.39	79.4 - 95.6	3.84	0.66
>23.09	40.74	22.4 - 61.2	90.91	81.3 - 96.6	4.48	0.65
>23.52	37.04	19.4 - 57.6	90.91	81.3 - 96.6	4.07	0.69
>23.86	33.33	16.5 - 54.0	90.91	81.3 - 96.6	3.67	0.73
>25.4	33.33	16.5 - 54.0	92.42	83.2 - 97.5	4.4	0.72
>26.46	29.63	13.8 - 50.2	92.42	83.2 - 97.5	3.91	0.76
>26.64	29.63	13.8 - 50.2	93.94	85.2 - 98.3	4.89	0.75
>26.96	25.93	11.1 - 46.3	93.94	85.2 - 98.3	4.28	0.79
>28.27	22.22	8.6 - 42.3	93.94	85.2 - 98.3	3.67	0.83
>29.14	22.22	8.6 - 42.3	95.45	87.3 - 99.1	4.89	0.81
>29.31	22.22	8.6 - 42.3	96.97	89.5 - 99.6	7.33	0.8
>30.91	18.52	6.3 - 38.1	96.97	89.5 - 99.6	6.11	0.84
>32.08	14.81	4.2 - 33.7	96.97	89.5 - 99.6	4.89	0.88
>33.07	14.81	4.2 - 33.7	98.48	91.8 - 100.0	9.78	0.86
>34.07	11.11	2.4 - 29.2	98.48	91.8 - 100.0	7.33	0.9
>35.29	7.41	0.9 - 24.3	98.48	91.8 - 100.0	4.89	0.94
>36.54	3.7	0.09 - 19.0	98.48	91.8 - 100.0	2.44	0.98
>38.02	3.7	0.09 - 19.0	100	94.6 - 100.0		0.96
>39.43	0	0.0 - 12.8	100	94.6 - 100.0		1

LS, Liver stiffness; PD, Progressive disease; CI, confidence Interval; +LR, positive likelihood ratio; -LR, negative likelihood ratio; *: the cut-off value has the highest sum of sensitivity and specificity. Bold values are values with statistical differences (P value<0.05).

### Development and Validation of the Nomogram

**Figure 3:**
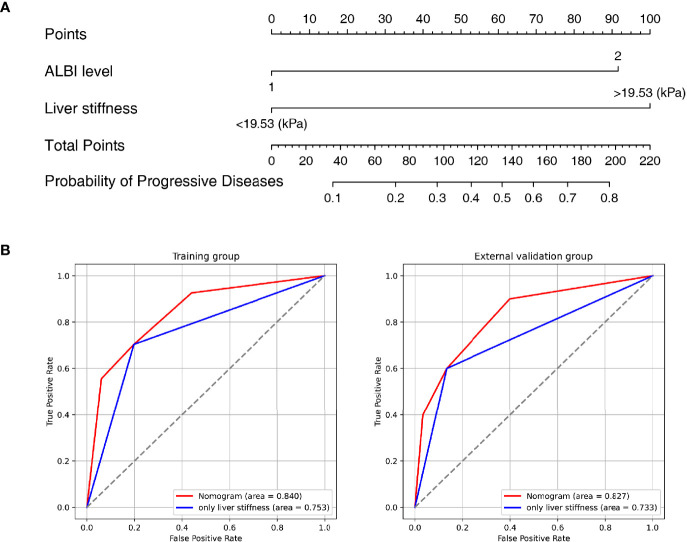
Nomogram and Receiver operating characteristic (ROC) curves analysis. **(A)** Nomogram for predicting probability of Progressive Diseases (PD) after therapy with anti–programmed cell death protein 1 (PD-1) plus lenvatinib. **(B)** ROC curves for 2 models in the training (left) and validation (right) set. Model based on baseline liver stiffness (LS) values of tumorous foci >19.53 kPa were shown in blue. Nomogram contains both baseline LS of tumorous foci and ALBI grade were shown in red.

**Figure 4:**
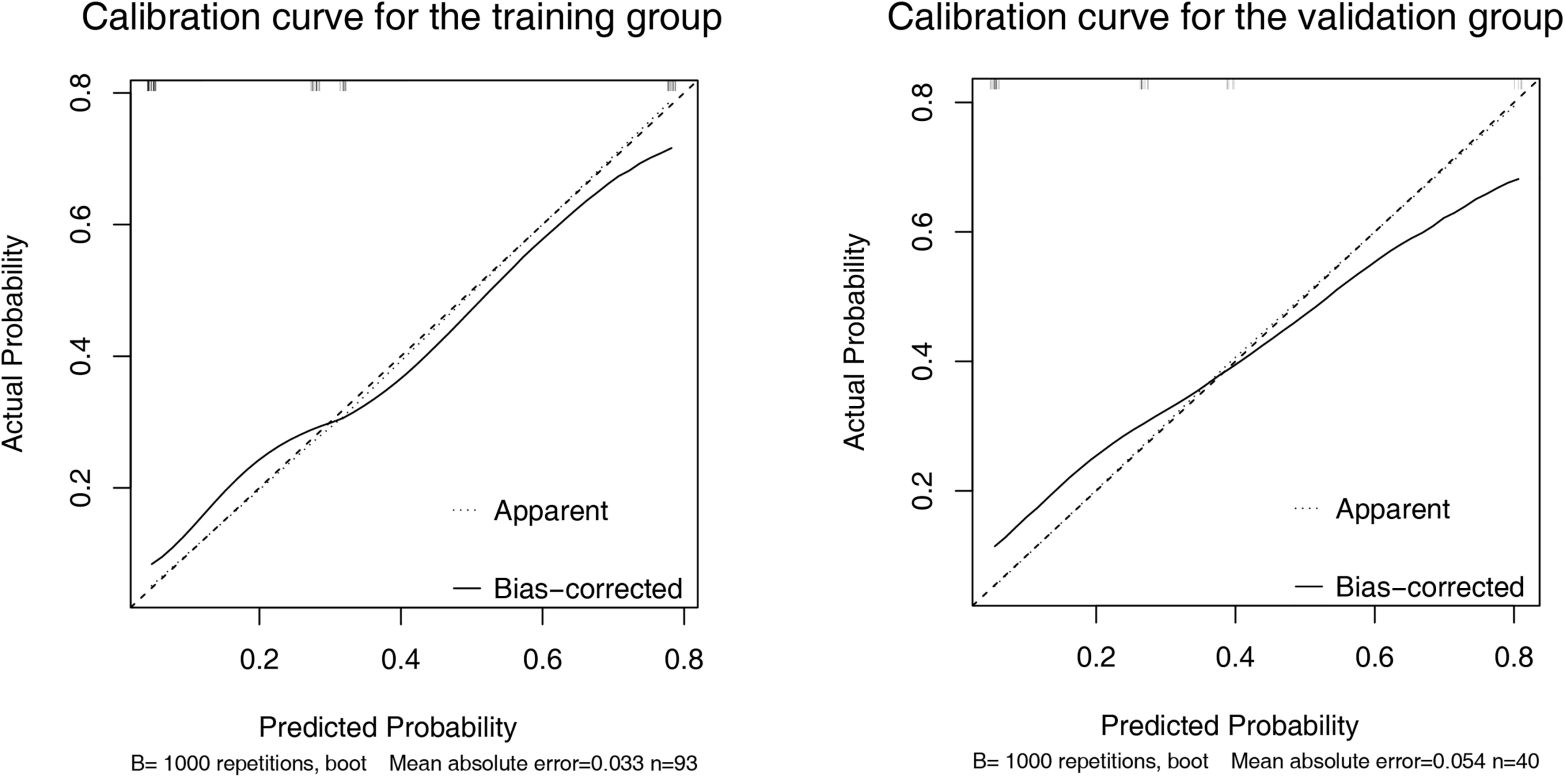
Calibration curve of the nomogram in the training **(A)** and validation **(B)** set. X-axis represents the nomogram predicted probability of Progressive Diseases (PD). Y-axis represents the actual probability of PD, and the diagonal dashed line (represent ideal) indicates the ideal prediction by a perfect model. Results were plotted *via* bootstrapping with 1000 resamples. The closer the bias-corrected calibration curve (solid line) is to the diagonal line, the higher the prediction accuracy of the model.

**Figure 5:**
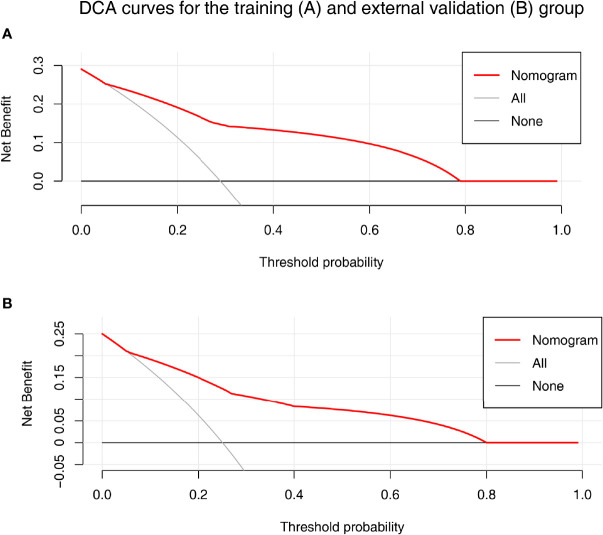
The decision curve analysis (DCA) for the nomogram in training **(A)** and validation **(B)** set. Nomogram contains both baseline LS of tumorous foci and ALBI grade were shown in red. Result showed that using the nomogram for PD prediction has more benefit than the treat-all-patients scheme (gray curve). A larger area under the decision curve suggested a better clinical utility.

A nomogram was established based on the results of multivariate logistic regression (**Figure 3A**). Variables of the nomogram included ALBI grade (grade 2) and baseline LS value of tumorous foci (>19.53kPa). Harrell’s C-index was 0.828 and 0.803 respectively (*P*>0.05) in the training and validation set. In the training set, the nomogram yielded an AUC of 0.840 (95%CI 0.750-0.931) with a sensitivity of 55.6% and a specificity of 93.9%. In the validation set, the nomogram exhibited an AUC of 0.827 (95%CI 0.673-0.980) with a sensitivity of 40.0% and a specificity of 96.7% (**Figure 3B**). Calibration curves (**Figure 4**) and Hosmer-Lemeshow test indicated good consistency between the nomogram-predicted probability of PD and the actual PD rate in both sets (*P*=0.548 and *P*=0.657). DCA demonstrated a higher net benefit of the nomogram than treated-all and treat-non strategy, indicating that treatment strategies based on our nomogram prediction have favorable clinical utility (**Figure 5**).

### Tumor Stiffness–Related Biological and Immune Characteristics in Patients With HCC of Nanfang Hospital Cohort

**Figure 6:**
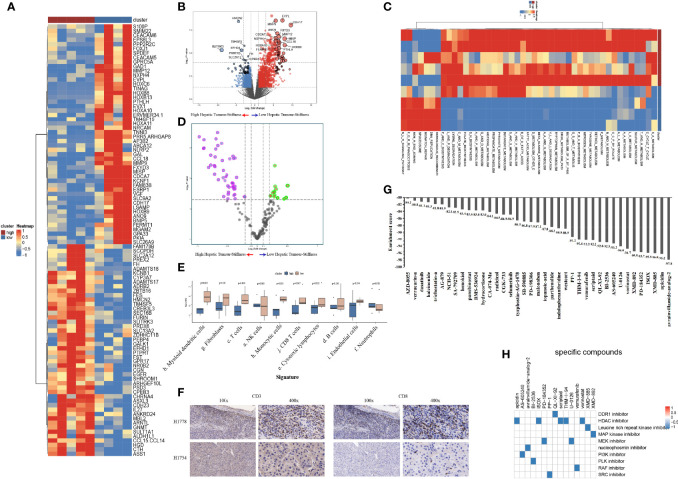
Biological functions and immune characteristics of tumors with high (n = 5) or low (n = 4) stiffness. **(A, B)** Heatmap **(A)** and volcano plot **(B)** comparing differences in gene expression between tumors with high and low stiffness. **(C, D)** Heatmap **(C)** and volcano plot **(D)** comparing differences in Kyoto Encyclopedia of Genes and Genomes (KEGG) pathways between tumors with high and low stiffness. **(E)** Boxplots of cell infiltration levels calculated by the Microenvironment Cell Populations-counter among tumors with high and low stiffness. **(F)** Representative micrographs of CD3 and CD8 protein expression in tumor samples with high and low stiffness, as detected by immunohistochemistry (H1778 was a patient with low stiffness liver tumor, and H1754 was a patient with high stiffness liver tumor). **(G)** Bar plot showing the enrichment score of each candidate compound in the connectivity map analysis. Compounds are sorted from right to left as the highest to lowest enriched, as assessed using an enrichment analysis. **(H)** Heatmap showing the mechanisms of the action (rows) of each compound (columns) from the connectivity map analysis. A total of 15 candidate drugs with absolute connectivity scores of <−90 were identified. Among them, 5 were histone deacetylase (HDAC) inhibitors.

**Figure 7:**
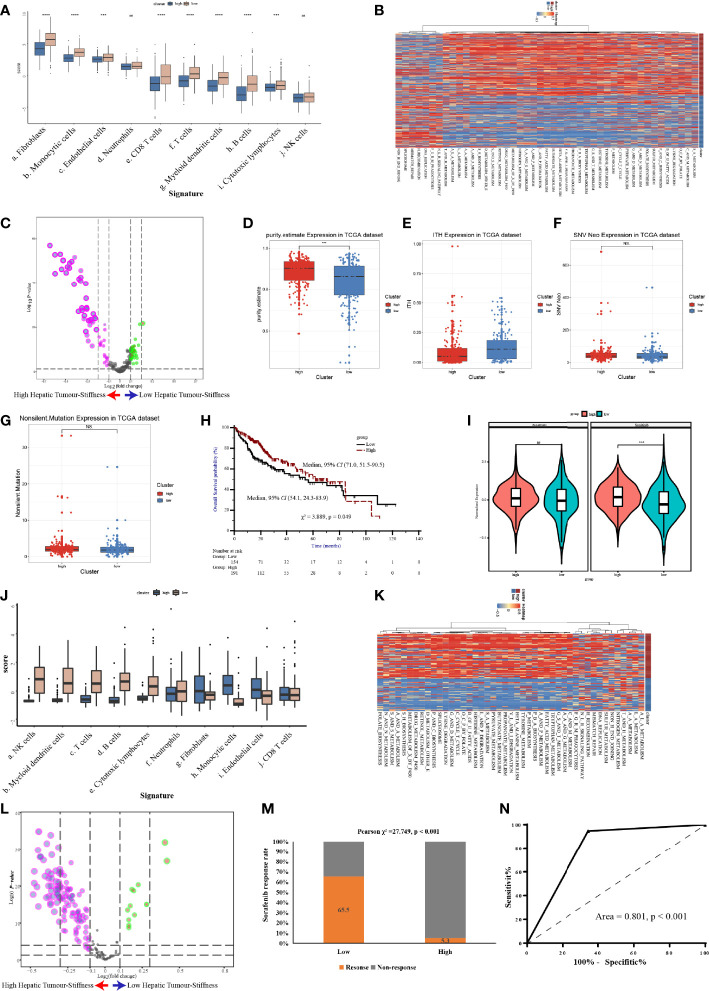
Validation of the high stiffness signature in TCGA-LIHC and GSE109211 datasets. **(A)** Boxplots of cell infiltration, as calculated by the Microenvironment Cell Populations-counter (MCP-counter), among tumors with high and low stiffness from The Cancer Genome Atlas Liver Hepatocellular Carcinoma (TCGA-LIHC) cohort. **(B, C)** Heatmap **(B)** and volcano plot **(C)** comparing differences in the Kyoto Encyclopedia of Genes and Genomes (KEGG) pathways between tumors with high and low stiffness in the TCGA-LIHC cohort. **(D–G)** Boxplots of tumor purity **(D)**, intratumoral heterogeneity (ITH) **(E)**, neoantigen **(F)**, and tumor mutation burden **(G)** in the 4 studied histone modification patterns of the TCGA-LIHC cohort. **(H)** Kaplan-Meier curves of overall survival in the TCGA-LIHC cohort according to tumor stiffness. **(I)** Violin plot of the estimated IC50 value of sorafenib and sunitinib calculated based on the Genomics of Drug Sensitivity in Cancer database among tumors with high or low stiffness in the TCGA-LIHC cohort. **(J)** Boxplots of cell infiltration levels calculated by the MCP-counter among tumors with high and low stiffness in the GSE109211 cohort. **(K, L)** Heatmap **(K)** and volcano plot **(L)** comparing differences in KEGG pathways between tumors with high and low stiffness in the GSE109211 cohort. **(M)** Bar charts summarizing the proportions of patients with different sorafenib responses across different tumor stiffness groups in the GSE109211 cohort. **(N)** Receiver operating characteristic curves of treatment response predictions of sorafenib according to the tumor stiffness signature in the GSE109211 cohort. NS means not-siginificant (without statistical difference); *** means p value < 0.001; **** means p value < 0.0001.

To explore the heterogeneity of biological and immune characteristics associated with tumor stiffness in HCC, we identified DEGs between tumors that were high stiffness (HS) versus low stiffness (LowS). A total of 483 tumor stiffness–related genes were found, 406 of which were upregulated in HS tumors, and the other 77 genes were more abundant in LowS tumors (**Figures 6A, B**). Kyoto Encyclopedia of Genes and Genomes (KEGG) analysis showed that the DEGs were enriched for multiple metabolism events (**Table S1**), indicating that metabolic differences might be the fundamental mechanisms underlying matrix stiffness effects on the tumor’s biological behavior. To visually show the differences in pathway activation between the two groups, we performed GSVA enrichment against the KEGG gene set. As shown on the heatmap and volcano plot (**Figures 6C, D**), the HS tumor group presented enrichment pathways predominantly associated with metabolism pathways, including KEGG_SELENOAMINO_ACID_METABOLISM, and KEGG_TAURINE_AND_HYPOTAURINE_METABOLISM, while the LowS tumor group was significantly enriched in pathways related to DNA damage repair (abbreviations of the pathways on the heatmaps are shown in **Table S2**). As for the immune landscape, the results of MCP-counter analysis showed that the LowS group had a relatively higher amount of immune cell infiltration, especially of dendritic cells, total T cells, and CD8+ T cells, suggesting that LowS tumors present with a hot microenvironment (an immune cell-dense microenvironment) phenotype (**Figure 6E**). We further characterized immune cell infiltration with IHC and found results consistent with the MCP-counter analysis (**Figure 6F**). Finally, we employed the Cmap tool to identify candidate drugs that might improve the efficacy of immunotherapy in patients with HS tumors. As shown in **Figures 7G, H**, 15 candidate drugs with absolute connectivity scores of <−90 were identified. Among them, 5 were histone deacetylase (HDAC) inhibitors, indicating that strategies to deactivate the HDAC pathway might be useful to promote infiltration of cytotoxic T cells into the microenvironment of HS tumors.

### Validation of the Distinct Biological and Immune Characteristics Associated With Tumor Stiffness in The Cancer Genome Atlas Liver Hepatocellular Carcinoma and GSE109211 Cohorts

To further confirm the molecular changes underlying HCC tumors with different stiffness levels in patients from Nanfang hospital, we used the NTP algorithm to distinguish tumor tissues in the TCGA-LIHC cohort and the GSE109211 cohort according to the DEGs found between the HS and LowS groups. In the TCGA-LIHC cohort, 204 (54.5%) patients were assigned into the HS group. Similarly, in the GSE109211 cohort, 80 (57.6%) patients belonged to the HS group. Like the results from the Nanfang hospital analysis, the GSVA and immune analyses of the TCGA-LIHC and GSE109211 cohorts demonstrated that the tumors of the HS group also represented a metabolic activation phenotype and showed an absence of immune cell infiltration (**Figures 7A–C, J–L**). Moreover, we obtained data for intratumoral heterogeneity (ITH), tumor purity, tumor mutation burden (TMB), and number of neoantigens from the study of Thorsson et al. (27) and compared these values between HS and LowS groups. Patients in the HS group exhibited less ITH and a higher tumor purity (**Figures 7D, E**). However, there were no significant differences in TMB or in the number of neoantigens between HS and LowS groups (**Figures 7F, G**). Intriguingly, survival analysis of patients in the TCGA-LIHC cohort showed significant prognostic differences among the two different tumor stiffness groups, with the HS signature being associated with a better prognosis (**Figure 7H**). Finally, we analyzed the relationship between the tumor stiffness associated gene and pathway signature we developed and the efficacy of anti-vascular therapy. In the TCGA-LIHC cohort, we used the “pRRophetic” package to predict treatment response to both sorafenib and sunitinib. As shown in **Figure 7I**, the LowS group was more sensitive to sorafenib and sunitinib, although the estimated IC50 was significantly different between the HS and LowS groups only for sunitinib. In addition, by analyzing the GSE109211 cohort, we found that sorafenib-sensitive patients were mainly concentrated in the LowS group, and the tumor stiffness signature had the ability to predict response to sorafenib with an AUROC value of 0.801 (**Figures 7M, N**).

## Discussion

In recent decades, immune checkpoint inhibitors—particularly antibodies targeting the PD-1/PD-L1 pathway—have gained popularity, becoming more commonly used in the clinic. However, with their widespread use came a gradual realization that they were effective in only a fraction of patients (10-30%), indicating it is urgently needed to develop robust predictors as useful tools for precise immunotherapy of advanced HCC (28, 29). In a previous study, we developed and validated a radiomics nomogram by incorporating pretreatment contrast-enhanced computed tomography images and clinical factors to estimate the efficacy of anti-PD-1 antibodies treatment in patients with advanced HCC (22). However, the development of a radiomics nomogram needs precise feature extraction of tumorous foci and professionals to carry out tedious machine learning, which greatly limits its application in our daily clinical practice. Therefore, more effective and convenient predictive tools are needed.

Previous studies have identified important HCC etiologies, like viral hepatitis, fatty liver disease, and alcoholic cirrhosis—each of which can induce fibrosis and lead to the development of HCC (30–32). Indeed, fibrosis is a verified factor that leads to HCC, and over 80% of patients with HCC have liver fibrosis (14, 33, 34). Accordingly, liver stiffness has been gradually accepted as an indicator of prognosis in patients with HCC. For example, Lee et al. found that LS values measured by 2D-SWE significantly predicted overall survival after radiofrequency ablation for HCC (14), while magnetic resonance elastography (MRE)-assessed LS has been highlighted as a potential radio-omics biomarker for predicting the prognosis of patients with chronic liver disease and HCC (13). However, no study has yet examined LS values as predictors for anti-PD-1 antibodies treatment efficacy. In the present report, we assessed the performance of LS values, as measured by SWE, for predicting response to therapy with anti-PD-1 antibodies in combination with lenvatinib in patients with advanced HCC. We demonstrated that a baseline tumor LS of >19.53 kPa was associated with higher rates of PD. We chose to test tumor LS values measured by SWE as a potential tool for predicting treatment efficacy of PD-1 inhibitor-based therapy for the following reasons: 1) with recent advances in ultrasound technology, various elastography techniques, including TE and SWE, have been confirmed to be effective tools for staging the degree of fibrosis (15–17); 2) in contrast to TE, SWE provides additional real-time information on tumorous foci, enabling a more detailed characterization that can help to predict the nature and behavior of the tumor (35); and 3) compared to TE, SWE shows a higher rate of reliable measurements and a similar predictive value for fibrosis, as determined in a meta-analysis of 13 studies (36).

To validate the predictive value of the data obtained from SWE, AUROCs were calculated for baseline tumor LS values in predicting PD after treatment with anti-PD-1 antibodies plus lenvatinib; we found that the AUROC was increased in both the training and validation sets compared with the normal liver tissue. Recently, Kim et al. reported that higher LS values, as assessed by MRE, are a potential biomarker for predicting poor overall survival and significant liver injury in patients with advanced HCC who were treated with sorafenib (13). This is in concordance with our current data, where tumor LS was identified as a strong predictor of treatment efficacy after anti-PD-1 antibodies in combination with lenvatinib. Furthermore, we have verified the predictive value of hepatic tumor stiffness by developing a nomogram, which showed favourable discrimination and calibration values.

The mechanism underlying the differential anti-PD-1 antibodies in combination with lenvatinib response associated with tumor stiffness in HCC remains unknown. This may be partly due to the dense extracellular matrix (ECM) that forms a barrier for T cells since the fibrotic state of desmoplastic tumors can cause immunosuppression through multiple mechanisms. First, it has been proposed that ECM may act as a physical barrier to CD8^+^ T cells infiltration into tumors. In addition to physical exclusion, matrix density and architecture could induce the localization and migration of T cells into the tumor stroma rather than into tumor cell nests (37). Furthermore, cellular components of tumor-associated fibrosis, particularly the cancer-associated fibroblasts (CAF), can have both direct and indirect effects on T cell infiltration and function (38). To explore the heterogeneity of biological and immune characteristics associated with tumor stiffness in HCC—and thus, potential explanations of the differential response—we identified DEGs between high- and low-stiffness tumor groups using scRNA-seq and IHC staining. We found that the DEGs that were enriched in high stiffness tumors were predominantly associated with metabolic pathways, while those enriched in low stiffness tumors were related to DNA damage repair, indicating that metabolic differences might drive the matrix stiffness–induced effects on the tumor’s biological behavior. Further analyses using the MCP-counter and IHC were performed to explore the immune landscape based on matrix stiffness. Our data showed that patients with low tumor stiffness had relatively higher immune cell infiltration, suggesting low LS is associated with a hot immune microenvironment phenotype, which might explain the differential responses to anti-PD-1–based therapy. In addition, the above molecular changes associated with tumors of different stiffnesses were further validated in the TCGA-LIHC and GSE109211 cohorts. Finally, we identified candidate drugs targeting the HDAC pathway as potentially useful strategies for promoting the infiltration of cytotoxic T cells into the microenvironment of high LS tumors, with the idea that this would enhance the efficacy of immunotherapies. Further experimental research focusing on the efficacy and safety of HDAC inhibitors in HCC patients with high stiffness liver tumors is needed to verify our conjecture.

Several limitations should be considered while interpreting our results. First, this is a retrospective study, which may have selection bias. The conclusions drawn from this study should be verified in larger prospective studies. Second, only 9 HCC samples were collected for RNA sequencing and IHC analysis, and thus, selection bias and confounding factors could not be eliminated. Third, several factors affecting the stiffness of liver tumors—e.g., MAFLD (metabolic associated fatty liver disease), ascites and jaundice, have not been analyzed in our current study. Therefore, additional multicenter, randomized controlled prospective studies are needed, specifically in patients with advanced HCC who receive a PD-1 inhibitor in combination with lenvatinib as the first-line regimen, to evaluate the predictive ability of tumor LS and the underlying mechanisms of LS-associated disease progression.

In conclusion, baseline LS values of tumorous foci by SWE prior to initiation of treatment with a PD-1 inhibitor plus lenvatinib were found to conveniently predict PD-1 inhibitor efficacy in patients with advanced HCC. It may be possible to apply these findings in the future for pretreatment stratification aimed at optimizing treatment outcomes in patients with advanced HCC. Metabolic differences and immune cell infiltration abundance may be the underlying mechanisms driving matrix stiffness effects on the tumor’s biological behavior.

## Data Availability Statement

The original contributions presented in the study are publicly available. This data can be found here: https://www.ncbi.nlm.nih.gov/,PRJNA816189

## Ethics Statement

The studies involving human participants were reviewed and approved by the Medical Ethics Committee of Nanfang hospital and the First Affiliated Hospital of Sun Yat-Sen University. The patients/participants provided their written informed consent to participate in this study.

## Author Contributions

(I) Conception and design: GY, FX, YS, JS, JC. (II) Administrative support: JC. (III) Provision of study materials or patients: XH, QL, RL, MZ, XC, GL, JC, JH. (IV) Collection and assembly of data: GY, QL, RL, XH, MZ, XC, GL, JC. (V) Data analysis and interpretation: GY, FX, YS, XH, JC. (VI) Manuscript writing: All authors. (VII) Manuscript revised: GY, FX, YS, QL, WF, XR, JSu, JC. (VIII) Final approval of manuscript: All authors.

## Funding

This study was supported by grants from the National Natural Science Foundation of China (82102879), the Natural Science Foundation of Guangdong Province (2021A1515012518), and the Postdoctoral Research Foundation of China (No. 2021M691468). The funding agencies had no role in the study design, data collection and analysis, decision to publish, or preparation of the manuscript.

## Conflict of Interest

The authors declare that the research was conducted in the absence of any commercial or financial relationships that could be construed as a potential conflict of interest.

The reviewer GW declared a shared parent affiliation with the author WF to the handling editor at the time of the review

## Publisher’s Note

All claims expressed in this article are solely those of the authors and do not necessarily represent those of their affiliated organizations, or those of the publisher, the editors and the reviewers. Any product that may be evaluated in this article, or claim that may be made by its manufacturer, is not guaranteed or endorsed by the publisher.
