# Supplementary material for: Hepatic Tumor Stiffness Measured by Shear Wave Elastography Is Prognostic for HCC Progression Following Treatment With Anti-PD-1 Antibodies Plus Lenvatinib: A Retrospective Analysis of Two Independent Cohorts
Source: Front Immunol. 2022 Jun 9;13:868809. doi: 10.3389/fimmu.2022.868809 (PMC9218245; doi:10.3389/fimmu.2022.868809)
Supplement: Supplementary file 2 [file Table_2.docx]

**Table S2. Abbreviations of the pathways from the heatmaps in Figures 5 and 6.**

| Full name | Abbreviations |
| --- | --- |
| KEGG_FC_GAMMA_R_MEDIATED_PHAGOCYTOSIS | F_G_R_M_PHAGOCYTOSIS |
| KEGG_HOMOLOGOUS_RECOMBINATION | H_RECOMBINATION |
| KEGG_DNA_REPLICATION | DNA_REPLICATION |
| KEGG_SPLICEOSOME | SPLICEOSOME |
| KEGG_NON_HOMOLOGOUS_END_JOINING | NON_H_END_JOINING |
| KEGG_NOD_LIKE_RECEPTOR_SIGNALING_PATHWAY | N_L_R_SIGNALING_PATHWAY |
| KEGG_MISMATCH_REPAIR | MISMATCH_REPAIR |
| KEGG_ALPHA_LINOLENIC_ACID_METABOLISM | A_L_A_METABOLISM |
| KEGG_SELENOAMINO_ACID_METABOLISM | S_A_METABOLISM |
| KEGG_TAURINE_AND_HYPOTAURINE_METABOLISM | T_AND_H_METABOLISM |
| KEGG_SULFUR_METABOLISM | SULFUR_METABOLISM |
| KEGG_ARACHIDONIC_ACID_METABOLISM | A_A_METABOLISM |
| KEGG_PANTOTHENATE_AND_COA_BIOSYNTHESIS | P_AND_C_BIOSYNTHESIS |
| KEGG_ONE_CARBON_POOL_BY_FOLATE | O_C_P_BY_FOLATE |
| KEGG_NICOTINATE_AND_NICOTINAMIDE_METABOLISM | N_AND_N_METABOLISM |
| KEGG_CYSTEINE_AND_METHIONINE_METABOLISM | C_AND_M_METABOLISM |
| KEGG_STARCH_AND_SUCROSE_METABOLISM | S_AND_S_METABOLISM |
| KEGG_LYSINE_DEGRADATION | LYSINE_DEGRADATION |
| KEGG_NITROGEN_METABOLISM | NITROGEN_METABOLISM |
| KEGG_LINOLEIC_ACID_METABOLISM | L_A_METABOLISM |
| KEGG_FOLATE_BIOSYNTHESIS | FOLATE_BIOSYNTHESIS |
| KEGG_ARGININE_AND_PROLINE_METABOLISM | A_AND_P_METABOLISM |
| KEGG_ALANINE_ASPARTATE_AND_GLUTAMATE_METABOLISM | A_A_AND_G_METABOLISM |
| KEGG_PYRUVATE_METABOLISM | PYRUVATE_METABOLISM |
| KEGG_CITRATE_CYCLE_TCA_CYCLE | C_CYCLE_T_CYCLE |
| KEGG_STEROID_HORMONE_BIOSYNTHESIS | S_H_BIOSYNTHESIS |
| KEGG_PHENYLALANINE_METABOLISM | P_METABOLISM |
| KEGG_GLYOXYLATE_AND_DICARBOXYLATE_METABOLISM | G_AND_D_METABOLISM |
| KEGG_BIOSYNTHESIS_OF_UNSATURATED_FATTY_ACIDS | B_OF_U_FATTY_ACIDS |
| KEGG_TYROSINE_METABOLISM | TYROSINE_METABOLISM |
| KEGG_DRUG_METABOLISM_OTHER_ENZYMES | D_METABOLISM_OTHER_E |
| KEGG_HISTIDINE_METABOLISM | HISTIDINE_METABOLISM |
| KEGG_ASCORBATE_AND_ALDARATE_METABOLISM | A_AND_A_METABOLISM |
| KEGG_METABOLISM_OF_XENOBIOTICS_BY_CYTOCHROME_P450 | METABOLISM_OF_X_BY_P450 |
| KEGG_DRUG_METABOLISM_CYTOCHROME_P450 | DRUG_METABOLISM_P450 |
| KEGG_RETINOL_METABOLISM | RETINOL_METABOLISM |
| KEGG_TRYPTOPHAN_METABOLISM | TRYPTOPHAN_METABOLISM |
| KEGG_BUTANOATE_METABOLISM | BUTANOATE_METABOLISM |
| KEGG_PROPANOATE_METABOLISM | PROPANOATE_METABOLISM |
| KEGG_BETA_ALANINE_METABOLISM | BETA_ALANINE_METABOLISM |
| KEGG_VALINE_LEUCINE_AND_ISOLEUCINE_DEGRADATION | V_L_AND_I_DEGRADATION |
| KEGG_GLYCINE_SERINE_AND_THREONINE_METABOLISM | G_S_AND_T_METABOLISM |
| KEGG_LIMONENE_AND_PINENE_DEGRADATION | L_AND_P_DEGRADATION |
| KEGG_FATTY_ACID_METABOLISM | FATTY_ACID_METABOLISM |
| KEGG_PRIMARY_BILE_ACID_BIOSYNTHESIS | P_B_A_BIOSYNTHESIS |
